# Association between dietary acid load and cancer risk and prognosis: An updated systematic review and meta-analysis of observational studies

**DOI:** 10.3389/fnut.2022.891936

**Published:** 2022-07-27

**Authors:** Ran Wang, Zhao-Yan Wen, Fang-Hua Liu, Yi-Fan Wei, He-Li Xu, Ming-Li Sun, Yu-Hong Zhao, Ting-Ting Gong, Hui-Han Wang, Qi-Jun Wu

**Affiliations:** ^1^Department of Clinical Epidemiology, Shengjing Hospital of China Medical University, Shenyang, China; ^2^Clinical Research Center, Shengjing Hospital of China Medical University, Shenyang, China; ^3^Department of Obstetrics and Gynecology, Shengjing Hospital of China Medical University, Shenyang, China; ^4^Department of Hematology, Shengjing Hospital of China Medical University, Shenyang, China

**Keywords:** dietary acid load, prognosis, risk, systematic review, cancer, meta-analysis

## Abstract

Epidemiological studies have suggested that dietary acid load (DAL) might be related to the risk and prognosis of cancer, whereas the evidence is contentious. Several high-quality observational studies have been published following a prior systematic review with only one study included. Consequently, we conducted an updated systematic review and meta-analysis to comprehensively investigate the relationship between DAL and cancer risk and prognosis. A systematic literature search was conducted in the PubMed, Embase, and Web of Science databases from inception to 26 October 2021. Summary relative risks (RRs) with 95% CIs were calculated using a random-effects model. Publication bias, subgroup, meta-regression, and sensitivity analyses were also conducted. Ten observational studies (six cohorts and four case–control studies) with 227,253 participants were included in this systematic review and meta-analysis. The summary RRs revealed a statistically significant associations between DAL and cancer risk (RR = 1.58, 95% CI = 1.23–2.05, *I*^2^ = 71.9%, *n* = 7) and prognosis (RR = 1.53, 95% CI = 1.10–2.13, *I*^2^ = 77.1%, *n* = 3). No evidence of publication bias was observed in the current analysis. Positive associations were observed in most subgroup analyses stratified by predefined factors, including region, study design, study quality, study population, participants’ gender, age of participants, cancer type, DAL assessment indicator, and adjustment of potential confounding parameters. No evidence of heterogeneity between subgroups was indicated by meta-regression analyses. The high DAL might be associated with an increased risk of cancer, as well as a poor prognosis of cancer. More high-quality prospective studies are warranted to further determine the associations between DAL and risk and prognosis for specific cancers.

## Introduction

Cancer is a leading cause of death and an important barrier to prolonging life (1). Globally, more than 19 million new cases of cancers were diagnosed, and nearly 10 million deaths from cancer occurred in 2020 (2). Most cancers were caused by a complex etiology such as environment, genetics, and lifestyle factors (3), and evidence had suggested that over 40% of cancer deaths could be prevented through changes in lifestyles, including diet (4). Due to the potential interaction between food and nutrients, the studies of dietary patterns or overall diet quality may better measure the impact of diet on health outcomes (5).

Dietary acid load (DAL) is one of the indexes to evaluate the quality of the whole diet, which provides more comprehensive information about the dietary intakes of subjects (6). It has been recently proposed that higher DAL, representing the consumption of diets characterized by a higher intake of meat and eggs and a lower intake of vegetables and fruits, could lead to changes or imbalances in blood pH and acid-base balance (7). DAL could be calculated through the potential renal acid load (PRAL), the net endogenous acid production (NEAP), the protein to potassium (Pro:K) ratio, and the net acid excretion (NAE), which are validated methods to assess DAL from dietary composition data (8, 9). Negative values of PRAL and lower values of NEAP, Pro:K, and NAE reflect alkaline-forming potential, whereas positive values of PRAL and higher values of NEAP, Pro: K, and NAE indicate acid-forming potential.

Experimental evidence has indicated that an acidic environment had a benign effect on the survival of cancer cells and promoted the invasion and metastasis of tumors (10, 11). The alkaline environment had the opposite effect on cancer cell survival compared with acidic environments (12). Several observational studies have also suggested that DAL is positively associated with some chronic diseases, such as metabolic syndrome (13) and type 2 diabetes (14). In 2016, Fenton and Huang (15) conducted a systematic review and found only one study focused on the association between DAL and cancer risk, which suggested null results. Interestingly, several epidemiological studies have published their results in recent years, but the findings have been controversial (16–25). For example, a large cohort study with 43,570 participants showed that consumption of high DAL food increased the risk of breast cancer (19). In contrast, a cohort study of 27,096 male smokers suggested a significant relationship between high DAL and an increased risk of bladder cancer (23).

To the best of our knowledge, there has been no updated systematic review and meta-analysis comprehensively verifying whether DAL plays a vital role in cancer risk and prognosis after the study of Fenton and Huang (15). Therefore, given the controversial findings as well as the current lack of high-level evidence of this issue, we conducted the present study to further understand and investigate the aforementioned topic.

## Methods

### Search strategy

This systematic review and meta-analysis were reported according to the Preferred Reporting Items for Systematic Reviews and Meta-Analyses guidelines (26) and the Meta-analysis of Observational Studies in Epidemiology group (27). PubMed, Embase, and the Web of Science databases were searched systematically to obtain studies published up to 26 October 2021 by two independent investigators (RW and ZYW). The following search keywords were utilized: (diet or dietary or diet dependent) and (acid or acid-base or NEAP or potential renal net acid load or DAL) and (cancer or neoplasms or oncology). Our search was completed by an additional manual search of reference lists of all the retrieved articles.

### Dietary acid load definitions

There were four ways to estimate DAL: (i) PRAL, which considered the absorption rates for dietary proteins and minerals, ionic dissociation, and sulfur metabolism (28); (ii) NEAP, which took into account the acidification of proteins and the alkalization of potassium (8); (iii) Pro:K that also involved animal proteins and potassium (8); and (iv) NAE that similar to PRAL, which further included estimated excretion of organic acids (12).

### Study selection and exclusion

To be included in this review, the following criteria were used for inclusion: (i) studies had an observational design, including cross-sectional, case–control, and cohort studies; (ii) studies assessing the relationship between DAL and cancer risk and prognosis; and (iii) studies recommending relative risk (RR), hazard ratio (HR), odds ratio (OR), or required data for an estimate. The studies were excluded for the following reasons: (i) studies that were not original research, including editors, case reports, and reviews; (ii) studies with randomized controlled or ecological design; and (iii) studies published in other languages instead of English.

### Data extraction and quality assessment

The studies that fulfilled all the inclusion criteria were qualitatively evaluated by two investigators (RW and ZYW), and any disagreements were settled by a discussion with a third investigator (QJW). The extracted data included the first author, the year of publication, country, design of studies included, number of cases, dietary assessment index, exposure categories, risk estimates, and adjusted variables. We assessed the quality of the articles according to the Newcastle–Ottawa Scale (NOS; 29). The NOS consisted of three fields: selection, comparability, and outcome. These studies received full marks in at least two categories of selection, comparability, or outcome assessment and were classified as low-risk bias (30, 31).

### Statistical analysis

In the meta-analysis, effect sizes for DAL were extracted from original studies, including standardized incidence ratio, HR, and RRs. The OR estimate and HR estimate were considered an approximation of the RR estimate (31). We calculated RR and 95% CI with a random-effects model (32) as a measure of the effect size for all the studies. A random-effects model accounted for variation between studies, as this can provide more conservative results than a fixed-effects model (33).

Heterogeneity in the relationship between DAL and cancer risk and prognosis across studies was quantified using *I*^2^ statistics. Cutoff points of ≤25, ≤50, ≤75, and >75% were used to indicate no, small, moderate, and substantial levels of heterogeneity, respectively, (34). To explore the sources of heterogeneity among studies, we conducted subgroup analyses and sensitivity analyses. Subgroup analyses were conducted based on region, study design, study quality, study population, gender, age, cancer type, DAL assessment indicator, and adjustments made for potential confounders, including body mass index, cigarette smoking, alcohol consumption, and physical activity. We also made a meta-regression model to identify potential sources of heterogeneity between subgroups. Sensitivity analysis was performed in which each study was eliminated from the study to evaluate the influence of that study (35). Publication bias was assessed by Begg’s test (36), Egger’s test (37), and visual inspection of funnel plots. A probability (*P*) value of <0.05 was considered statistically significant. All the analyses were conducted using Stata version 11.2 software (StataCorp, College Station, TX, United States).

## Results

### Search results, study characteristics, and quality assessment

**FIGURE 1:**
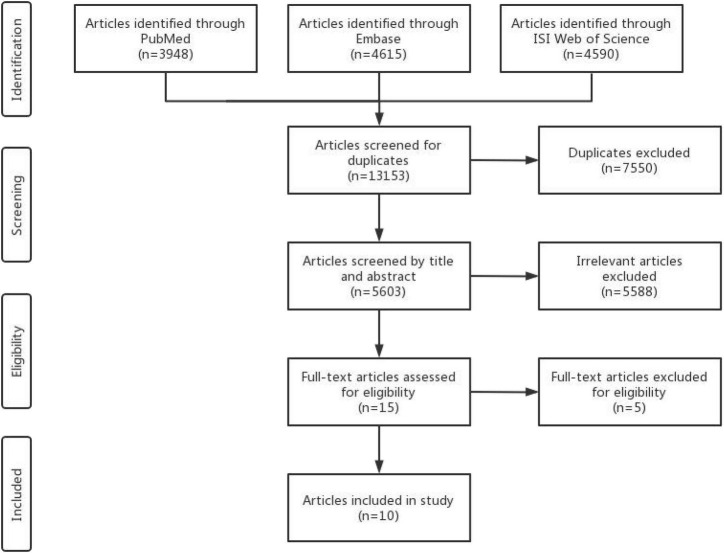
Flowchart of study selection.

The search strategy retrieved 13,153 articles from databases, of which 5,605 articles remained after removing the 7,550 duplicate articles. After the initial screening based on titles or abstracts, 5,588 studies were excluded, leaving 17 studies included. Of these, 5 articles (38–42) were further eliminated because of the duplicated study population and incomplete results. The final selection yielded 10 articles (16–25; 7 studies for cancer risk and 3 studies for cancer prognosis; Figure 1) included in the meta-analysis.

Seven studies focused on cancer risk were published between 2005 and 2021 (Table 1). Among them, four were case–control studies (17, 18, 20, 21), and three were cohort studies (19, 22, 23). Three studies were performed in Asia (17, 18, 21), two in North America (19, 22), one in South America (20), and one in Europe (23), respectively. DAL had been assessed using the PRAL and NEAP methods in five studies (17, 19–22), NAE in two studies (22, 23), and Pro:K in three studies (17, 20, 22). The included articles were assessed by dietary intake through the Food Frequency Questionnaire (FFQ) and the Diet History Questionnaire (DHQ). Potential confounders were adapted for age (*n* = 6), energy intake (*n* = 6), family history of cancer (*n* = 6), smoking status (*n* = 6), and body mass index (*n* = 5; Table 2). Five studies (lung, glioma, colorectal, breast, and pancreatic cancers) indicated a relationship between higher DAL intake and an increased risk of cancer (17–20, 22), whereas two studies (breast and bladder cancers) demonstrated a null association (21, 23).

**TABLE 1:** Characteristics of studies included in the systematic review and meta-analysis.

First author (ref), year, Country	Study design	Type of cancer	No. of case/event	No. of participants	Dietary assessment/ index	Exposure categories	Risk estimates (95%CI)
Hejazi et al. (16), Iran	Cohort study	NA	1,502	48,691	FFQ/PRAL	Q5 vs. Q2 PRAL	HR: 1.04 (0.89–1.22)
Milajerdi et al. (18), Iran	Case-control study	Glioma	128	384	FFQ/Pro: K	T3 vs. T1 Pro: K	OR: 3.05 (1.04–8.91)
Ronco et al. (20), Uruguay	Case-control study	Lung	843	2,309	FFQ/PRAL, NEAP	Q4 vs. Q1 PRAL NEAP	OR: 0.99 (0.64–1.52) OR: 2.22 (1.52–3.22)
Shi et al. (22), United States	Cohort study	Pancreatic	337	95,708	DHQ/PRAL, NEAP	Q4 vs. Q1 PRAL NEAP	HR: 1.73 (1.21–2.48) HR: 1.64 (1.14–2.36)
Nasab et al. (17), Iran	Case-control study	Colorectal	259	499	FFQ/PRAL, NEAP, Pro: K	T3 vs. T1 PRAL	OR: 4.82 (2.51–9.25)
Wu et al. (24), United States	Cohort study	Breast	295	2,950	24-h dietary recalls/PRAL, NEAP	Q4 vs. Q1 PRAL NEAP	HR: 1.30 (0.87–1.94) HR: 1.54 (1.04–2.29)
Wu et al. (25), United States	Cohort study	Breast	517	3,081	24-h dietary recalls/PRAL, NEAP	Q4 vs. Q1 PRAL NEAP	HR: 2.15 (1.34–3.48) HR: 2.31 (1.42–3.74)
Park et al. (19), United States	Cohort study	Breast	1,882	43,570	FFQ/PRAL, NEAP, Pro: K, NAE	Q4 vs. Q1 PRAL	HR: 1.21 (1.04–1.41)
Safabakhsh et al. (21), Iran	Case-control study	Breast	150	300	FFQ/PRAL, NEAP	T3 vs. T1 PRAL NEAP	OR: 1.00 (0.29–3.36) OR: 0.92 (0.25–3.36)
Wright et al. (23), Finland	Cohort study	Bladder	446	27,096	FFQ/NAE	Q5 vs. Q1 NAE	RR: 1.15 (0.86–1.55)

CI, confidence interval; DHQ, diet history questionnaire; FFQ, food frequency questionnaire; HR, Hazard Ratio; NA, not report; NAE, renal net acid excretion; NEAP, net endogenous acid production; OR, Odds Ratio; PRAL, potential renal acid load; Pro:K, Protein:Potassium (K); Q, quartile or quintile; RR, Relative Risk; and T, tertile.

**TABLE 2:** Adjustment potential confounders of included studies.

First author (ref), year	Adjustment for potential confounders in the primary analysis
Hejazi et al. (16)	Age, sex, BMI, smoking, alcohol, opium, wealth score, physical activity, dietary fat, carbohydrate, fiber intake, history of CVD, COPD, renal failure, diabetes
Milajerdi et al. (18)	Age, sex, energy intake, marital status, smoking, family history of cancer, physical activity, supplement use, disease duration, high-risk residential area, history of exposure to the radiographic X-ray, history of head trauma, duration of cell phone use, history of allergy, history of hypertension, exposure to chemicals, drug use, frequent fried food intake, frequent use of barbecue, canned foods and microwave, high-risk occupation, dietary intakes of polyunsaturated fatty acids, sodium, calcium, selenium, vitamin C, vitamin E, vitamin B6, folic acid, BMI
Ronco et al. (20)	Age, residence, family history of cancer in first degree, BMI, smoking intensity, alcohol status, “Mate” intake, tea intake, energy, total fiber, total carotenoids, lignans, flavonols, glutathione, vitamin C, vitamin E, animal-based iron, total heterocyclic amines
Shi et al. (22)	Age, sex, smoking status, history of diabetes, alcohol intake, BMI, family history of pancreatic cancer, dietary fiber, carbohydrate, energy intake from diet
Nasab et al. (17)	Age, comorbidity, cancer family history, common ways of cooking, level of salt intake, physical activity, calcium supplement use
Wu et al. (24)	Age at diagnosis, race/ethnicity, education level, intervention group, menopausal status at baseline, total calorie intake, alcohol intake, physical activity, BMI, number of comorbidities, tumor stage, tumor size, estrogen and progesterone receptor status, tamoxifen use, radiotherapy, chemotherapy
Wu et al. (25)	Age at diagnosis, race/ethnicity, education level, intervention group, menopausal status at baseline, total calorie intake, alcohol intake, smoking status, pack-years, physical activity, BMI, tumor stage, tumor size, estrogen and progesterone receptor status, tamoxifen use, radiotherapy, chemotherapy
Park et al. (19)	Age, race, household income, physical activity, pack-years of smoking, BMI, alcohol consumption, total energy intake, recent mammogram screening, stronger family history of breast cancer, breastfeeding history, parity, postmenopausal hormone therapy, age at menopause, multivitamin use
Safabakhsh et al. (21)	BMI, education, marital status, menopause status, socioeconomic status, alcohol use, smoking, vitamin supplements and medication uses, medical history, history of hormone replacement therapy, time of oral contraceptive use, age at first menarche, time since menopause in postmenopausal women, weight at age 18 years old, number of children, length of breastfeeding, family history of breast cancer, energy intake
Wright et al. (23)	Age, energy intake, number of years of smoking, cigarettes/day, intervention assignment

BMI, body mass index; COPD, chronic obstructive pulmonary disease; CVD, cardiovascular disease; “Mate” is the name of the staple infusion in Uruguay, made from the Ilex paraguariensis herb.

Table 1 demonstrates the characteristics of the cancer prognosis studies (16, 24, 25), which were referred to as cohort studies. Of them, two studies were undertaken in North America (24, 25) and one study was undertaken in Asia (16). PRAL was assessed in all the studies, whereas NEAP was applied in two studies (24, 25). Dietary intake was evaluated through FFQ and 24-h dietary recall in all the included studies. Risk estimates were adjusted for body mass index (*n* = 3), smoking status (*n* = 3), physical activity (*n* = 3), and age at diagnosis (*n* = 2; Table 2). Two cohort studies indicated a significant relationship between higher DAL (represented by NEAP) intake and poor survival among patients with breast cancer (24, 25), whereas one cohort study demonstrated a null association (16).

The information on quality assessment is given in Tables 3, 4. Five cohort studies (16, 19, 22, 24, 25) were graded as low risk, whereas only one cohort study (23) was graded as high risk (Table 3). For the item of “control for important factor or additional factor,” four studies (16, 23–25) were not awarded two stars since these studies adjusted for less than two important confounder factors. For the classification of “outcome,” two studies (22, 23) were not assigned full stars because of the inadequacy of the follow-up rate of cohorts. Most included case–control studies (75%) were at high risk (Table 4). For the “selection” classification, three studies (17, 18, 20) were not assigned full stars. For the item of “control for important factor or additional factor,” one study (17) was not awarded two stars since these studies had adjusted for less than two important confounder factors in their analysis. For the classification of “exposure,” two studies (18, 20) were not assigned full stars because there was a significant difference in the response rate between cases and controls.

**TABLE 3:** Methodological quality of cohort studies included in the systematic review and meta-analysis.

First author, reference, publication year	Selection				Comparability	Outcome			Risk of bias^d^
	Representativeness of the exposed cohort	Selection of the unexposed cohort	Ascertainment of exposure	Outcome of interest not present at start of study	Control for important factor or additional Factor^a^	Assessment of outcome	Follow-up long enough for outcomes to occur^b^	Adequacy of follow-up of Cohorts^c^	
Hejazi et al. (16)	*	*	*	*	*	*	*	*	Low risk
Shi et al. (22)	*	*	*	*	**	*	*	–	Low risk
Wu et al. (24)	*	*	*	*	*	*	*	*	Low risk
Wu et al. (25)	*	*	*	*	*	*	*	*	Low risk
Park et al. (19)	*	*	*	*	**	*	*	*	Low risk
Wright et al. (23)	*	*	*	*	*	*	*	–	High risk

*A study could be awarded a maximum of one star for each item except for the item Control for important factor or additional factor. The definition/explanation of each column of the Newcastle-Ottawa Scale is available from (http://www.ohri.ca/programs/clinical_epidemiology/oxford.asp).

^a^This project receives a maximum of two stars. One star can be obtained by adjusting for total energy intake and another star can be obtained by adjusting for other important confounding factors.

^b^A cohort studies with follow-up > 5 years or cohort studies with prognosis > 1 year were eligible for one star.

^c^A cohort study with a follow-up rate > 75% is assigned one star.

^d^Studies that obtained full scores in at least two domains were considered to have a low risk of bias, other situations were considered as high risk.

**TABLE 4:** Methodological quality of case–control studies included in the systematic review and meta-analysis.

First author, reference, publication year	Selection				Comparability	Exposure			Risk of bias^c^
	Adequate definition of cases	Representativeness of cases	Selection of control subjects	Definition of control subjects	Control for important factor or additional Factor^a^	Exposure assessment	Same method of ascertainment for all subjects	Non-response Rate^b^	
Milajerd et al. (18)	*	*	–	*	**	*	*	–	High risk
Ronco et al. (20)	*	*	–	*	**	*	*	–	High risk
Nasab et al. (17)	*	*	–	*	*	*	*	*	High risk
Safabakhsh et al. (21)	*	*	*	*	**	*	*	*	Low risk

^*^A study could be awarded a maximum of one star for each item except for the item Control for important factor or additional factor. The definition/explanation of each column of the Newcastle-Ottawa Scale is available from (http://www.ohri.ca/programs/clinical_epidemiology/oxford.asp).

^a^This project receives a maximum of two stars. One star can be obtained by adjusting for total energy intake and another star can be obtained by adjusting for other important confounding factors.

^b^One star is assigned if there is no significant difference in the response rate between control subjects and cases by using the chi-square test (*P* > 0.05).

^c^Studies that obtained a full scores at least two domains were considered to have a low risk of bias, other situations were considered as high risk.

### Association of dietary acid load with cancer risk

**FIGURE 2:**
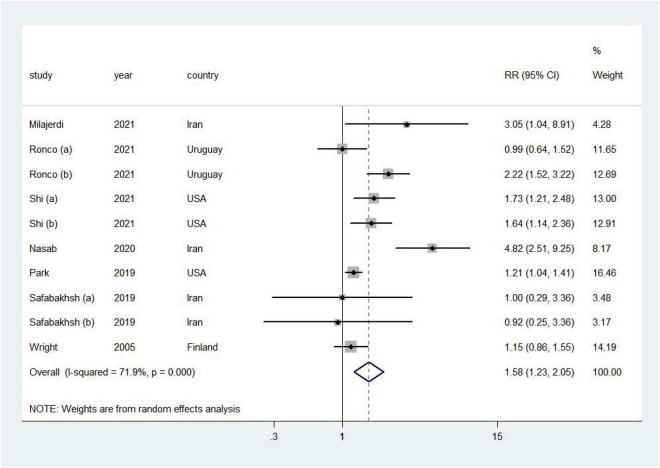
Forest plot (a random-effects model) of the association between DAL and cancer risk (highest vs. lowest). Squares indicate study-specific relative risk (RR), where the size of the square reflects the study-specific statistical weight; horizontal lines indicate the 95% CI; and diamonds denote the summary RR with 95% CI.

Higher DAL was associated with a 58% increased risk of cancer (RR = 1.58, 95% CI = 1.23–2.05, *I*^2^ = 71.9%; Figure 2). No publication bias was discovered (Supplementary Figure 1; Egger’s *P* = 0.21 and Begg’s *P* = 0.47).

Positive associations were found in most subgroup analyses (Table 5). Notably, in the stratified analysis, we observed significant positive associations in studies in non-Asia (RR = 1.41, 95% CI = 1.14–1.76), age of participants ≥50 years (RR = 1.60, 95% CI = 1.21–2.11), breast cancer (RR: 1.20, 95% CI = 1.03–1.40), and pancreatic cancer (RR = 1.69, 95% CI = 1.31–2.18). Furthermore, the risk of cancer incidence increased by 57% (RR = 1.57, 95% CI = 1.03–2.41) and 83% (RR = 1.83, 95% CI = 1.36–2.47) by high PRAL and NEAP, respectively. Additionally, meta-regression analysis revealed that there was no evidence of heterogeneity between these subgroup analyses.

**TABLE 5:** Summary risk estimates of the association between dietary acid load and risk of cancer (highest vs. lowest).

	No. of study	RR (95%CI)	*I^2^ (%)*	*P* ^1^	*P* ^2^
Overall	7	1.58 (1.23, 2.05)	71.90	<0.01	
Subgroup analyses					
Region					0.149
Asia	3	2.16 (0.92, 5.06)	63.50	0.042	
Non-Asia	4	1.41 (1.14, 1.76)	66.60	0.012	
Age					0.869
<50	2	1.51 (0.68, 3.32)	24.10	0.268	
≥50	5	1.60 (1.21, 2.11)	79.50	<0.01	
Sex					0.152
Men	2	1.36 (0.86, 2.18)	79.70	<0.01	
Women	2	1.20 (1.03, 1.40)	0.00	0.880	
Both	3	2.30 (1.45, 3.66)	67.90	0.025	
Cancer type					0.858
Breast cancer	2	1.20 (1.03, 1.40)	0.00	0.880	
Pancreatic cancer	2	1.69 (1.31, 2.18)	0.00	0.838	
Glioma	1	3.05 (1.04, 8.91)	N/A	N/A	
Lung cancer	1	1.49 (0.68, 3.30)	N/A	N/A	
Bladder cancer	1	1.15 (0.86, 1.54)	N/A	N/A	
Colorectal cancer	1	4.82 (2.51–9.25)	N/A	N/A	
Study design					0.372
Cohort study	3	1.86 (1.05, 3.28)	75.10	<0.01	
Cross-sectional study	4	1.35 (1.12, 1.62)	45.50	0.138	
Study population*					0.149
<Median	3	2.16 (0.92, 5.06)	63.50	0.042	
≥Median	4	1.41 (1.14, 1.76)	66.60	0.012	
Study quality					0.382
Low risk	3	1.91 (1.12, 3.24)	83.60	<0.01	
High risk	4	1.38 (1.13, 1.67)	24.40	0.259	
DAL assessment indicator					0.812
PRAL	5	1.57 (1.03, 2.41)	80.50	<0.01	
NEAP	3	1.83 (1.36, 2.47)	18.00	0.295	
Pro: K	1	1.15 (0.86, 0.55)	N/A	N/A	
NAE	1	3.05 (1.04, 8.91)	N/A	N/A	
Adjust body mass index					0.429
Yes	5	1.49 (1.17, 1.89)	57.30	0.022	
No	2	2.28 (0.56, 9.29)	93.50	<0.01	
Adjust alcohol drinking					0.429
Yes	5	1.49 (1.17, 1.89)	57.30	0.022	
No	2	2.28 (0.56, 9.29)	93.50	<0.01	
Adjust cigarette smoking					0.241
Yes	4	2.46 (0.86, 7.07)	88.30	<0.01	
No	3	1.44 (1.14, 1.83)	57.80	0.027	
Adjust physical activity					0.233
Yes	3	2.49 (0.88, 7.02)	89.30	<0.01	
No	4	1.44 (1.14, 1.83)	52.00	0.051	

CI, confidence interval; NA, not applicable; RR, relative risk.

^1^*P*-value for heterogeneity within each subgroup.

^2^*P*-value for heterogeneity between subgroups with meta-regression analysis.

*The median study population for the analysis of DAL (highest vs. lowest) is 1,404.

In sensitivity analyses, we sequentially removed one study; in turn, the pooled RR did not change substantially. Our sensitivity analysis showed that the RR for cancer ranged from a low of 1.50 (95% CI = 1.16–1.95, *I*^2^ = 68.4%) after removing the study by Ronco et al. (20) to a high of 1.68 (95% CI = 1.23–2.29, *I*^2^ = 69.5%) after removing the study by Park et al. (19; Supplementary Figure 2).

### Association of dietary acid load with cancer prognosis

**FIGURE 3:**
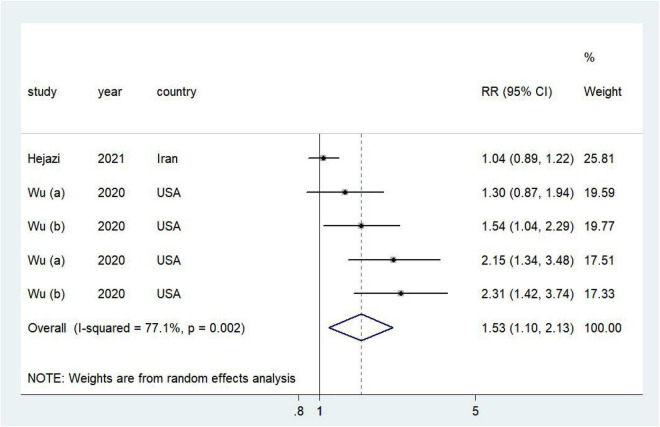
Forest plot (a random-effects model) of the association between DAL and cancer prognosis (highest vs. lowest). Squares indicate study-specific relative risk (RR), where the size of the square reflects the study-specific statistical weight; horizontal lines indicate the 95% CI; and diamonds denote the summary RR with 95% CI.

Higher DAL was associated with a poor prognosis of cancer (RR = 1.53, 95% CI = 1.10–2.13, *I*^2^ = 77.1%; Figure 3). No publication bias was discovered (Supplementary Figure 3; Egger’s *P* = 0.02 and Begg’s *P* = 0.09). In sensitivity analyses, we sequentially removed one study; in turn, the pooled RR did not change substantially. Our sensitivity analysis showed that the RR for cancer ranged from a low of 1.41 (95% CI = 1.01–1.98, *I*^2^ = 74.6%) after removing the study by Wu et al. (25) to a high of 1.62 (95% CI = 1.05–2.48, *I*^2^ = 82.7%) after removing the study by Wu et al. (24; Supplementary Figure 4).

## Discussion

To the best of our knowledge, the present review is the most comprehensive study reporting the relationship between DAL and cancer risk and prognosis. Findings from this systematic review and meta-analysis indicated that higher DAL might be an unfavorable factor for cancer risk and prognosis. These findings were consistently detected in numerous subgroups and sensitivity analyses.

Our findings are inconsistent with the previous systematic review, which included articles published before April 2015, and concluded that DAL was overall not significantly associated with an increased risk of cancer (15). However, this systematic review included only one study comprising 27,542 participants and 446 bladder cancers (23). Our systematic review and meta-analysis further included nine studies involving 227,253 participants published during the last 3 years (16–22, 24, 25). Of note, six low-risk studies were included in the present systematic review and meta-analysis (16, 19, 21, 22, 24, 25). Furthermore, numerous subgroup analyses and meta-regression analyses were conducted based on study characteristics and confounding factors.

In the subgroup analysis stratified by region, we only observed positive associations in studies carried out in the non-Asia region. This phenomenon could partly be attributed to the different DAL scores in patients with cancer from diverse regions. For example, when investigating 1,882 patients with breast cancer in the United States, it was found that the mean value was 2.25 for the PRAL score (19), whereas Safabakhsh et al. (21) reported that the mean value was –26.1 for the PRAL score based on 150 patients with breast cancer in Iran. Furthermore, a Western dietary pattern characterized by a high score of PRAL was associated with an increased risk of patients with cancer (43, 44).

The subgroup analyses suggested that DAL was positively associated with the risk of cancer in participants of age ≥50 years. Indeed, Frassetto et al. found that increasing age was associated with indicative of a progressively worsening low-level metabolic acidosis, and the changes seemed to be the most striking starting at about age 50 years (45). In addition, potential long-term effects of acidogenic diets are further compounded by the reduction of renal function typically from aging (45, 46). However, for the risk of cancer in participants at the age ≥50 years, more studies are warranted, mainly due to the presence of the high heterogeneity of these results.

Compared to the results of PRAL, the risk of cancer was considered to be substantially higher in NEAP. Both the PRAL and NEAP are approximate to DAL and highly correlated (*r* = 0.9; 8). However, the assessment of PRAL may be imprecise due to the error in the measurement of minerals or the protein intakes with low or high ranges (9, 47). In fact, Ronco et al. proposed that NEAP was found to be a better predictor of breast cancer risk than PRAL (20). One explanation is that PRAL relies on more information from the dietary database, which means that it may be more susceptible to confounding factors. Therefore, future studies should focus more on the accuracy of PRAL calculations.

Results of our subgroup analyses demonstrated that DAL increased the risk of breast and pancreatic cancers. PRAL is inversely correlated with the consumption of vegetables, while phytochemicals contained in vegetables may contribute to decreasing the level of the epidermal growth factor receptor (48, 49), which is known to be a major growth-stimulating factor exclusively in breast cancer (50). Furthermore, metabolic acidosis is found to reduce circulating adiponectin levels by inhibiting the transcription of the adiponectin gene (51); both the experimental and epidemiological studies have suggested a high level of adiponectin against the risk of pancreatic cancer (13, 52). In addition, we have previously found that a higher intake of red meat and dairy was statistically related to an increased risk of breast and pancreatic cancers (53–55). However, due to the limited number of studies, we yielded a null association between DAL and other cancers. Therefore, more prospective cohort studies of a specific cancer are needed to clarify these issues.

Several studies have indicated that consumption of high DAL dietary might be linked with a worse prognosis among patients with cancer (24, 25). Wu et al. (24) indicated that higher DAL was related to breast cancer-specific mortality and total mortality. Furthermore, Wu et al. (25) also found the same trend among 3,081 United States patients with breast cancer. Hejazi et al. (16), however, suggested that DAL was unrelated to the overall survival of cancer. They might miss an association between DAL and cancer survival because of unmeasured confounding and dietary changes. Of note, since dietary information was not updated during follow-up, they could not account for any changes in dietary consumption over time.

Although there was no evident mechanism to explain the relationship between DAL and cancer risk, several consensuses have been proposed. First of all, metabolic acidosis caused by DAL could promote cancer. Acid-base imbalance had been shown to regulate molecular activities, including insulin growth factor-1 (IGF-1; 56, 57) and osteoclast activation (58, 59), which may serve as intermediaries for cancer occurrence and promotion (60–62). In addition, acid-producing diets were often high in animal and processed proteins and low in fruits and vegetables, which were associated with a higher carcinogenic effect (63–65). The evidence also showed that DAL reduces circulating adiponectin (66), and both the experimental and epidemiological studies (13, 51, 52, 67) had shown that it played a role in the occurrence of cancer.

Regarding cancer prognosis, several studies existed to interpret this phenomenon. Metabolic acidosis had been shown to stimulate cancer metastasis in cell and animal models (68–70). In addition, metabolic acidosis depleted endogenous bicarbonate levels, which neutralize acids. A cross-sectional study showed that lower bicarbonate levels were associated with loss of muscle mass and reduced body function (32). As the precursors of bases, potassium (71), magnesium (72), and calcium (73) could inhibit the metastasis and the growth of cancer cells.

The principal strengths were that the present study was the most comprehensive systematic review to estimate the relationship between DAL and the risk and prognosis of cancer. We conducted a rigorous literature search to include all the pertinent studies. In consideration of study features and main adjustments for confounding variables, subgroup, sensitivity, and meta-regression analyses were conducted to probe into possible sources of heterogeneity. In addition, most of the selected articles had a low risk after using the NOS to evaluate the quality of all the included literature. Nevertheless, some limitations of this study should be recognized. First of all, measurement and recall bias in the assessment of dietary intake were inevitable. The calculation of PRAL, NEAP, NAE, and Pro:K based on self-reported data was collected by FFQ, DHQ, and 24-h dietary recalls. However, the majority of the included studies used valid and reliable FFQ, and it had been proved that FFQ could be more precise in assessing the association between diet and diseases (74). Second, the estimation methods of DAL had not been unified (8, 9). PRAL and NEAP were widely recognized and used, while Pro:K and NAE were seldom used, suggesting that one of the estimation methods could be used as the main calculation and the other three methods could be used as a sensitivity analysis in future studies. Third, we only located observational studies that fitted inclusion criteria, which means a large space for future research on DAL and cancer incidence and prognosis, especially in terms of prognosis. The studies on cancer prognosis were mainly concentrated on breast cancer, while other types of cancer had not been covered. Fourth, even though several confounding factors were considered, the included studies cannot rule out the possibility that unmeasured factors might have contributed to these associations.

In summary, the current systematic review and meta-analysis revealed that a higher DAL was associated with an increased risk and poor prognosis for cancers. Further large-scale prospective studies were warranted to explore the role of DAL in different cancers.

## Data availability statement

The raw data supporting the conclusions of this article will be made available by the authors, without undue reservation.

## Author contributions

RW, H-HW, and Q-JW conceived the study. RW, F-HL, Y-HZ, T-TG, and Q-JW contributed to the design. RW, Z-YW, and Q-JW collected the data, cleaned the data and checked the discrepancy, and analyzed the data. RW, Z-YW, Y-FW, H-LX, M-LS, Y-HZ, T-TG, H-HW, and Q-JW interpreted the data. All the authors have interpreted the data, read the manuscript, and approved the final vision of the manuscript.
