# Supplementary material for: Association between dietary acid load and cancer risk and prognosis: An updated systematic review and meta-analysis of observational studies
Source: Front Nutr. 2022 Jul 27;9:891936. doi: 10.3389/fnut.2022.891936 (PMC9365077; doi:10.3389/fnut.2022.891936)
Supplement: Supplementary file 1 [file Table_1.DOCX]

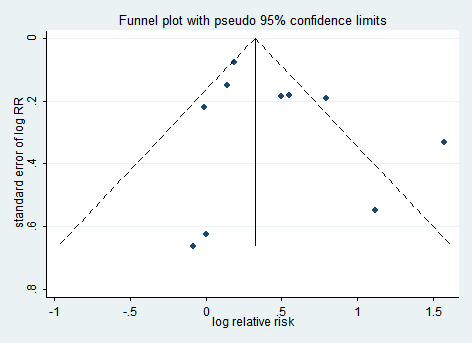


**Supplementary Figure 1.** Funnel plots for DAL (highest versus lowest) and risk of cancer.


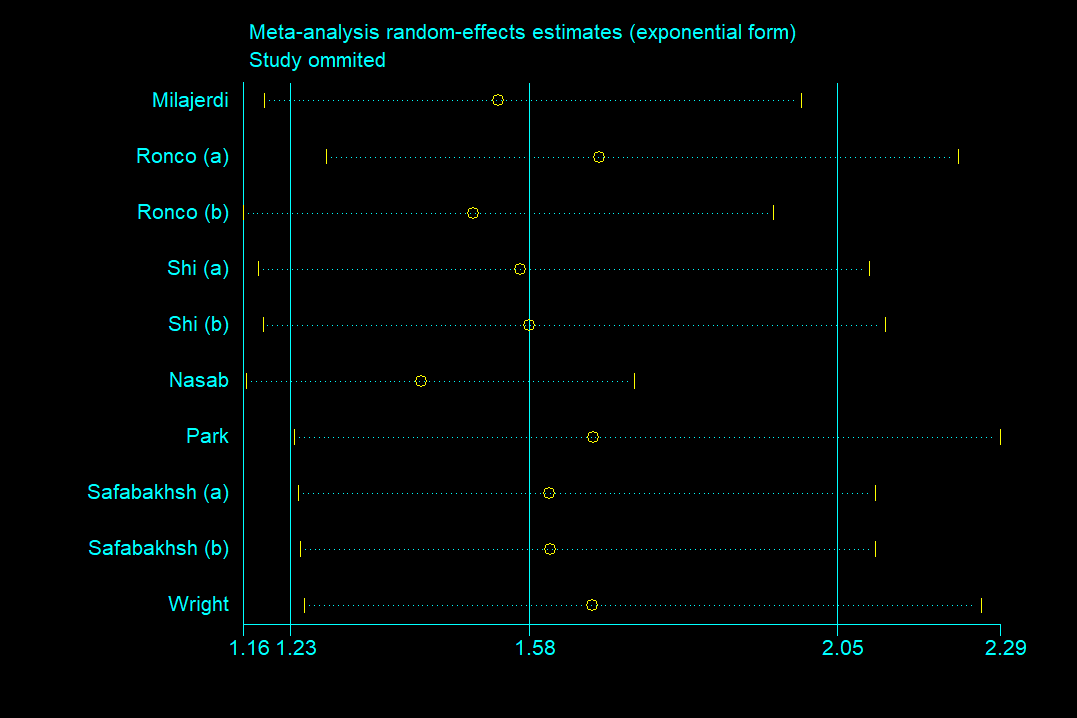


**Supplementary Figure 2.** Sensitivity analysis of meta-analysis of DAL (highest versus lowest) and risk of cancer by excluding each study in sequence.


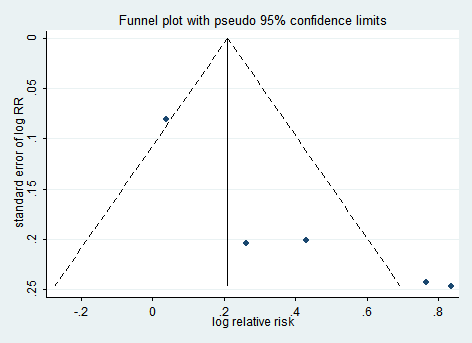


**Supplementary Figure 3.** Funnel plots for DAL (highest versus lowest) and prognosis of cancer.


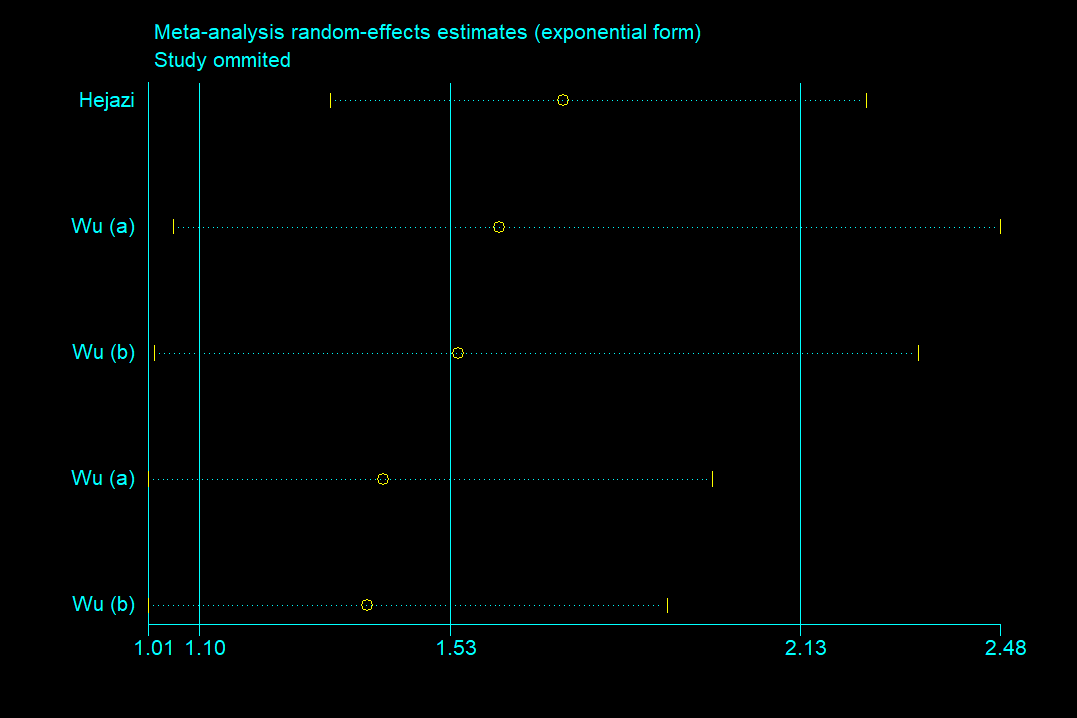


**Supplementary Figure 4.** Sensitivity analysis of meta-analysis of DAL (highest versus lowest) and prognosis of cancer by excluding each study in sequence.
